# Precursors and outcomes of work engagement among nursing professionals—a cross-sectional study

**DOI:** 10.1186/s12913-021-07405-0

**Published:** 2022-01-04

**Authors:** Terje Slåtten, Gudbrand Lien, Barbara Rebecca Mutonyi

**Affiliations:** Inland School of Business and Social Science, Inland Norway University of Applied Sciences, Campus Lillehammer, 2604 Lillehammer, Norway

**Keywords:** Work engagement, Turnover intention, Job satisfaction, Service quality, Collaborative climate, Organizational culture, Nursing professionals, Hospitals

## Abstract

**Background:**

Health services organizations must understand how best to lower nursing professionals’ turnover intentions, and increase their job satisfaction and the quality of care provided to patients. This study aims to examine whether work engagement (WE) is a significant predictor of the achievement of these preferred organizational goals. The study also aims to examine whether organizational culture and organizational climate can manage the WE of nursing professionals and indirectly contribute to the accomplishment of the preferred organizational goals.

**Methods:**

In detail, a cross-sectional questionnaire survey study was conducted through a convenience sampling of a total of *N* = 164 nurses, from four Norwegian public hospitals. Structural equation modeling was employed in testing the hypothesis in the conceptual model, using Stata software. Furthermore, mediation analyses were achieved through use of the “medsem” package in the Stata software, in testing whether the proposed direct and indirect effects were statistically significant, and the type of mediation found.

**Results:**

The three key findings from this study are: i) WE of nursing professionals was found to be positively related to service quality of care (β = 0.551) and job satisfaction (β = 0.883). Job satisfaction fully mediates the relationship between WE and turnover intention and in itself explains almost 60% (*R*^2^ = 0.59) of turnover intention; ii) nursing professionals’ perception of organizational culture (β = 0.278) and collaboration climate (β = 0.331) were both directly related to their WE; and iii) WE fully mediates the relationship between organizational culture/climate and service quality of care and job satisfaction. Moreover, WE partially mediates the relationship between collaborative climate and job satisfaction.

**Conclusions:**

The WE of nursing professionals is highly correlated to their job satisfaction. WE and turnover intentions are (fully) mediated by job satisfaction. Employers should therefore focus on improving the job satisfaction of nursing professionals. The WE of nursing professionals is a common key factor for such improvement. Consequently, leaders and managers should continuously manage nursing professionals’ WE, focusing on such areas as organizational culture and climate, because WE is an effective means of enabling multiple desirable outcomes for hospital organizations.

**Supplementary Information:**

The online version contains supplementary material available at 10.1186/s12913-021-07405-0.

## Background

Research has shown that a sustainable human resource base in healthcare organizations is a major challenge. For instance, issues such as motivation, retention and moral have created significant concern for healthcare organizations as well as for policy makers [1].

The difficulty in handling this major challenge of having a sustainable human resource base is especially relevant when considering nurses, who are characterized as an important foundation of human resource capital in healthcare organizations [2]. It has been demonstrated that healthcare organizations have experienced ongoing challenges of high turnover rates among nurses [3]. Such turnover has several negative impacts such as lower levels of service quality provided to patients [4], lower patient satisfaction [5], lower productivity [6], negative effect on morale and an increase in the level of turnover intentions [7]. Consequently, it has become essential for hospital organizations to identify factors that have the potential to contribute positively to achieving desired organizational goals, such as lowering turnover intentions among nurses as well as being able to increase (or maintain) their job satisfaction and level of service quality provided to patients. As in Gupta et al., this study suggests work engagement (WE) as a positive key element in achieving organizational goal achievement [1].

This study aims to examine whether the WE of nursing professionals has the potential to function as a core factor in the achievement of multiple desirable outcomes for hospital organizations. WE relates to nursing professionals’ perception of experiencing a positive response to the work they are performing [1] and has been proposed as an important factor for organizations to focus on [8]. Today, healthcare organizations are confronted with a double-edged problem: retention of their employees and simultaneously engaging their employees in their work role [8]. In a recent systematic review of WE in nursing, Keyko et al. maintained that WE is a strategic tool for addressing crucial challenges pertaining to healthcare [9]. Similarly, Bargagliotti argued that in the twenty-first century, positive nurses’ WE is essential for nurses’ personal initiative, and for health organizations’ profitability and efficiency [10].

Previous research on WE among nurses has linked WE to several issues, such as, but not limited to, affective commitment [1] and organizational attractiveness [2]. Specifically, in their extensive systematic review on WE, Keyko et al. identified a total of 77 influencing factors, such as leadership and autonomy, and 17 outcomes such as job performance associated with WE [9]. Furthermore, previous research has revealed that when WE is not properly cultivated in a health organization, it can result in lower levels of service quality provided to patients [4]. Moreover, studies show that the healthcare work environments of nurses can have devastating outcomes on nurses’ WE, such as reduced productivity [6] and reduced work morale [7]. In addition, the systematic review by Keyko et al. uncovered various knowledge gaps in the literature on nursing staff’s WE and introduced a job-demand resource model that aimed to motivate further research on nurses’ WE in healthcare organizations [9]. The extensive review by Keyko et al. [9] motivated the following four specific aims in the present study.

First, Keyko et al. argued for research focusing on nurses’ WE in relation to hospital patients [9]. This study follows this explicit recommendation by examining service quality of care (SQC) provided to patients as one type of outcome of nurses’ WE. By doing this, this study contributes to the WE literature by providing new knowledge of the importance and value of fostering WE in healthcare organizations [1, 2, 9]. Second, based on their review and regarding research identifying antecedents and outcomes related to nurses’ WE, Keyko et al. revealed that most research on nurses’ WE is dominated by North American studies, limiting our understanding of WE in other populations and settings [9]. Consequently, in responding to the recommendation to expand our current understanding of the role of nurses’ WE, this study examines nurses’ WE in a Norwegian hospital context and thus examines a Scandinavian population and setting. As such, this study contributes new empirical knowledge and adds to the current discussion on the potential differences found in geographical or cultural settings [1, 8, 10]. Third, and as mentioned, although Keyko et al. identified 77 factors that influence nurses’ WE, the findings reveal that no study included in the review had previously empirically examined how organizational culture is linked to nurses’ WE [9]. This is surprising because the “culture of an organization provides boundaries and guidelines that help members of the organization to know the correct way to behave towards each other, and how to perform their work tasks” ([11], p. 2711). According to Slåtten et al. [2, 12], healthcare organizations need to foster a culture and climate that focuses on health employees. Recently, internal market-oriented culture (IMOC) has been proposed as one type of culture that healthcare organizations should develop and cultivate in meeting challenges pertaining to WE [2, 12]. As such, Slåtten et al. calls attention to the underexplored research that empirically explores IMOC as a driver for WE in healthcare organizations [2, 12]. This study responds to the call of Keyko et al. and Slåtten et al. to address the knowledge gap by examining the empirical relationship between IMOC and individual nurses’ WE [2, 9]. Fourth, in their review, Keyko et al. also recommended that future research on nurses’ work engagement should include more advanced statistical analysis techniques that improve the overall quality of the research results [9]. This study addresses this recommendation by examining both precursors and outcomes of nurses’ WE in the same research model and thus offers an opportunity to perform more complex and complete statistical tests of both direct and mediating effects related to nurses’ WE.

Considering the abovementioned four aims, this paper contributes to extending and deepening previous research on nurses’ WE and thus contributes theoretically as well as suggesting practical implications to “enable development of initiatives that enhance work engagement and its outcomes within the current health care context” ([9], p. 143).

## Conceptual model

**Fig. 1:**
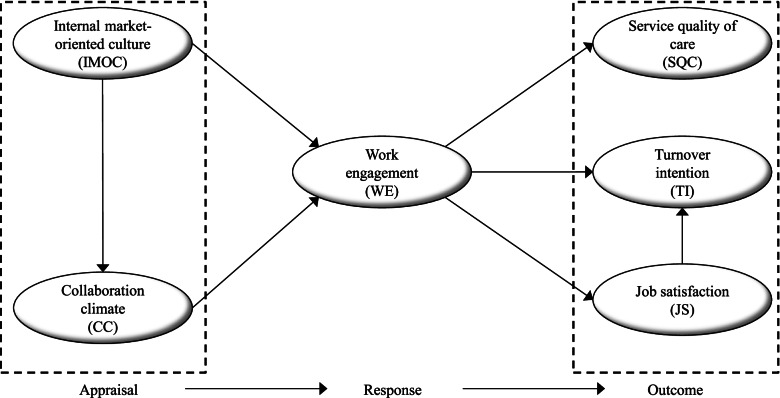
Conceptual model to examine precursors and outcomes of WE

Based on the discussion above and the forthcoming discussion on the study’s theoretical framework, this study proposes a conceptual model (Fig. 1). In Fig. 1, the six main constructs of this study are grouped into three categories: *Appraisal*, *Response* and *Outcome*. These categories are connected in a specific directional manner as shown by the arrows. As seen in Fig. 1, the two constructs under *Appraisal* IMOC and CC are proposed to be related to the *Response* variable WE. Furthermore, the conceptual model proposes three types of *Outcomes*: SQC, JS and TI. The *Response* construct WE is proposed to be related to SQC, JS and TI. In the following sections, each of the six constructs and their hypothesized relationships, as shown in Fig. 1, are further elaborated in detail.

## Theoretical framework

### Work engagement

The concept of WE originated from positive psychology, which focuses on the positive resources and strengths of humans, rather than their limits [13]. Specifically, in this study, WE is defined as “a positive, fulfilling, work-related state of mind” ([14], p. 74). Moreover, this positive, work-related state of mind embraces three interconnected types of feelings, namely, absorption, vigor and dedication. Absorption is about employees’ level of interest and engrossment or immersion of employees in their work such that the sense of time is lost [15]. Vigor reflects the workers’ level of energy and mental resilience while performing their work. Dedication describes how involved employees are in the sense of significance, inspiration and challenge [15]. It is important to note that WE is a “state of mind” ([14], p. 74). Defining WE as a state of mind and not a trait of a person means that WE, compared with the relative stability of a trait of a person, is dynamic. This dynamic feature implies that all “ingredients” of the internal resource pool of individual employees’ WE are potentially changeable and something that can fluctuate, both positively and negatively within a certain timeframe.

In this study, as indicated in the previous discussion and visualized in Fig. 1, WE is, as a response variable, studied as an individual-level resource. WE is heterogeneously distributed among individual employees in organizations, and is a variable that can be described as a motivational-like variable with the potential to improve organizational outcomes positively [16]. In the next section, three outcomes of WE are proposed.

#### The outcomes of work engagement

The individual-level resource WE is suggested to be linked to three different types of outcomes, namely, (i) turnover intention (TI), (ii) job satisfaction (JS) and (iii) service quality of care (SQC). All outcomes of WE represent desirable organizational goals that healthcare organizations want to achieve.

##### Turnover intention

In this study, turnover intentions (TI) embrace nurses’ psychological responses to conditions in organizations [17] and consequently their reflection about whether they should stay with the organization that employs them. It is important to note that TI focuses on intended intentions to leave a job on a personal/worker level. As such, TI reflects an important first cognitive step in the decision-making process [18] about leaving an organization.

Previous research has suggested that WE is negatively related to TI. When employees experience a positive state of mind characterized by vigor, dedication and absorption, referring to those elements embraced by the concept of WE, this should lead to more favorable evaluations and thoughts about their workplace and thus be associated with decisions or intentions not to leave the organization in which they are employed. Previous research on nurses supports the existence of a negative relationship between WE and TI. For example, in their study, Moloney et al. [19], using 2876 nurses working in New Zealand as respondents, found that nurses’ WE is negatively linked to the TI of their organization. Rodwell et al. [20] also found a negative link between nurses’ WE and nurses’ TI in their study of 459 nurses across Australia. Similarly, Wan et al. [6] found a significant negative relationship between WE and TI for 778 nurses employed in a hospital in China. Consistent with the aforementioned research findings, this study is expected to find a similar negative relationship between nurses’ WE and TI among those nurses included in this study (referring to nurses from Norway). Therefore, the following hypothesis is proposed:

- **Hypothesis 1:** *WE is significantly and negatively related to TI*

##### Job satisfaction

According to Tomietto et al., healthcare organizations are requesting strategies for how they can best cultivate a challenging factor such as JS [16]. In this study, WE is suggested to be one such strategy to achieve JS. Therefore, as seen in Fig. 1, JS is proposed to be one outcome of nurses’ WE. In this study, JS refers to the “extent to which employees [nurses] like their jobs” ([21], p. 13). JS is a cognitive concept that refers to nurses’ global assessment of their job [22].

Although JS has generally been included in several previous studies on nurses, it seems that relatively few of these have examined the specific link between WE and JS. For example, in their extensive review of antecedents and effects of nurses’ WE, Keyko et al. [9] only reported four previous studies examining this link. Of these four studies, two identified a significant relationship between WE and JS, one found an insignificant relationship between WE and JS, and another was qualitative in nature. Consequently, there is a need for more research on this topic with a specific focus on the domain of nursing. In general, it seems that previous research most often has identified a positive relationship between WE and JS [23]. It is reasonable to assume a similar pattern and relationship between these concepts among nurses. Consequently, when positively experienced, the three “ingredients” of WE should lead employees to have more favorable thoughts and feelings about their job. It is difficult to imagine a situation where an employee has a high level of WE and at the same time, dislikes/hates his/her job and thus has low JS. Consistent with previous research [23] on this topic, this study assumes that nurses’ WE is positively related to their JS. This reasoning leads to the second hypothesis in this study:

- **Hypothesis 2a:** *WE is significantly and positively related to JS*

Previous research has found that the JS of nurses is directly linked to TI among nurses [16]. However, in this study, it is also suggested that JS functions as a mediator between WE and TI. This represents a complementary “route” to TI compared with what is proposed in Hypothesis 1. In addition, to have a direct impact on TI, it is also assumed that there is some inner mechanism in humans that explains why there exists a relationship between WE and TI. In this study, this inner mechanism in humans refers to JS, which does not arise by itself or occur in isolation. JS is always triggered or created by someone or something. In this study, “something” refers to nurses’ WE. As discussed in the second hypothesis, previous research on WE suggested its impact on JS [9, 23]. Based on this, when nurses experience a positive WE, their JS will be positively affected. Furthermore, when JS increases because of an increase in WE, it is reasonable to assume that this will also lower the level of TI among nurses. In the study by Tomietto et al. [16], the authors conceptualize JS as one potential mediator between nurses’ WE and nurses’ TI. Nevertheless, the authors did not complete any empirical test of whether JS was operating as a mediator. Consequently, and to the best of the authors’ knowledge, this study is a pioneering study in empirically examining whether JS is operating as a mediator between WE and TI. The aforementioned reasoning can be expressed by this formal hypothesis:

- **Hypothesis 2b:** *The relationship between WE and TI is mediated by JS*

##### Service quality of care

In this study, SQC is studied as an organizational objective, because the best level of SQC to patients is a highly desirable organizational goal for hospital organizations to achieve [4]. Nurses make a considerable contribution to the total “service package” regarding SQC [2, 9, 11]. According to Chen et al., this group of frontline workers “tend to have the longest and closest contact with patients” ([24], p. 1). In this study, the concept of SQC refers to nurses’ perceptions of the overall quality of services provided. Although this is a subjective view in contrast to an objective way of capturing the content of SQC, it follows from how previous research has often studied SQC both within and outside healthcare and other contexts [2, 25]. Moreover, previous research also suggests that there exists a “psychosocial closeness” between providers’ and receivers’ perception of service quality [26]. Consequently, it is assumed in this study that nursing professionals are capable of considering whether their level of quality of services is of high or low standard or lies within a zone that “customers generally perceive as acceptable” ([27], p. 208). The assumption about the association is formulated by the following hypothesis:

- **Hypothesis 3:** *WE is positively related to service SQC*

#### Precursors to work engagement

In Fig. 1, precursors to WE are suggested to be (1) collaboration climate (CC) and (2) IMOC. Consequently, the following discussion is focused on whether the appraisal of CC and IMOC promotes or causes a response in the individual-level resources of employees referred to as WE in Fig. 1. Note that in this study, both CC and IMOC are studied as organizational-level resources.

##### Collaboration climate

An organizational climate is about employees’ shared perceptions of their organization. These shared perceptions of climate vary from strongly negative to strongly positive. The literature emphasizes the significance and value for organizations in focusing on organizational climate. For example, Kieft et al. found that “it is important to develop and maintain collaborative working relationships with professionals, including those in their own field” ([28], p. 5). Consequently, there are good reasons to include collaboration climate (CC) and how frontline employees perceive and appraise this organizational-level resource. However, in an organization, there are a variety of alternative aspects to focus on when studying a CC. Therefore, it is necessary to identify aspects that are both relevant and specific for the phenomenon in focus. Consequently, “climate is best regarded as a specific construct having a referent” ([29], p. 1278). The literature suggests several ways to study organizational CC. For example, D’Amour et al. suggest four ways to analyze cooperation in healthcare organizations [30]. In this study, CC is about two work-related relationships regarding interdepartmental collaboration in health organizations. Specifically, CC refers to frontline employees’ perception of interdepartmental (i) conflict and (ii) connectedness, both of which are suggested in previous research [31, 32]. The first climatic aspect of “conflict” of CC is about whether there exist tensions, caused by inconsistency regarding actual and desired responses between departments in the organization. The second climatic aspect, “connectedness,” focuses on whether there are formal and informal contacts across departments in the organization. This latter aspect reveals nursing professionals’ perceptions of whether departments are operating dependently or independently in relation to each other. These two aspects of CC represent and reflect nurses’ perceptions of the supportive work environment or what alternatively can also be labeled as the internal service climate in an organization [29].

According to the job-demands–resources (JD–R) model, WE is fostered by job resources [33]. In this study, CC represents this organizational level of resources in the JD–R model that promotes or fosters WE among frontline employees. According to Wan et al., “a supportive work environment … offers various resources to foster employees’ willingness to dedicate their efforts and abilities to job tasks” ([6], p. 1334). Although it seems that no study has empirically examined the link between CC and WE specifically, previous research has found that the work environment found in CC, in general, is positively associated with WE. For example, the work environment has been found to be associated with WE among nurses [34]. Furthermore, research has found that perceptions of organizational climate are linked to employees’ attitudes [35]. Consequently, based on the JD–R model and findings in previous research, there are good reasons to expect CC to be positively associated with WE. Therefore, the following hypothesis is proposed:

- **Hypothesis 4:** *CC is positively related to WE*

##### Internal market-oriented culture

In this study, and as mentioned above, IMOC is studied as an organizational-level resource. Organizational culture is, according to Banaszak-Holl, said to “pervade all aspects of organizational life” ([36], p. 462). Organizational culture is a “stable element deeply rooted in employees’ mentality” ([37], p. 585). Moreover, organizational culture embraces norms that “provide the rules for behavior” ([38], p. 2). Of the different components an organizational culture consists of, norms and behavior are the two most observable components [39]. In this study, organizational culture refers to nursing professionals’ perception of norm-based behavior regarding the IMOC in the organization. As such, and as noted in Slåtten et al., IMOC “focuses on more tangible or visible aspects of organizational culture that frontline employees of hospitals experience or observe daily” ([12], p. 160). The basic idea with the concept of IMOC is to treat employees in organizations as customers. Parallel to external customers, it is important to treat these internal customers (referring to employees) in the best possible way. Consequently, IMOC focuses on employees’ perception of whether managers’ norm-based behavior in the organization is oriented toward satisfying the needs and wants relevant to employees’ working conditions [12]. Three systems constitute the norm-based behavior concept of IMOC: (i) internal-market intelligence generation; (ii) internal-intelligence dissemination; and (iii) response to internal intelligence [40]. The three systems are closely connected and represent a logical flow of information from the first to the third system. The first system relates to management-related activities to collect information regarding the needs and wants of employees. The second system relates to management interpretation and understanding of employees’ needs and wants. Finally, the third system relates to the willingness and capability of the management in an organization to take steps to perform real actions and actively do what is necessary to satisfy the needs and wants of their employees. All systems of IMOC are interconnected. However, system three (referring to “response to internal intelligence”) is probably the part that employees are most likely to experience or observe in their day-to-day work and thus most prominently brings IMOC to “life” in the organization.

Organizational culture “strongly influences employee behaviors” ([39], p. 1) and thus is an employee-impacting instrument to create the desirable and necessary behavior in an organization. Previous research within healthcare organizations has found organizational culture to be associated with such areas as job satisfaction, leadership behavior, turnover intention and organizational attractiveness [2, 12, 38]. Regarding this and specifically referring to IMOC, Slåtten et al. argues that “IMOC is a type of organizational culture affecting frontline employees” ([12], p. 161). The core of IMOC is about management’s ability to satisfy needs and wants with a specific focus on employees’ work roles. With this in mind, it is reasonable to assume that when IMOC is perceived by employees as something good, it should positively influence employees’ vigor, absorption and dedication, which are all embraced in the concept of employees’ WE. The association between IMOC and WE is also supported in the JD–R model. The JD–R model highlights that different resources in the work environment can promote or act as motivational factors for nursing professionals’ dedication and efforts (or what this study refers to as WE) to perform work tasks [33]. As previously mentioned, this study has studied IMOC as an organizational-level resource. Therefore, based on the JD–R model and previous research, IMOC should have an impact on WE. This reasoning leads to the following hypothesis:

- **Hypothesis 5a:** *IMOC is positively related to WE*

Although IMOC is expected to be directly associated with WE, it is also assumed that this relationship is mediated through the concept of organizational climate, represented as CC in this study. Accordingly, it is proposed that two “routes” exist from IMOC to WE. In the literature, the concepts of climate and culture are often suggested to be closely related. Regarding conceptual closeness, Carlucci noted that “culture and climate are similar concepts” ([37], p. 585). Although at first sight they seem to be rather similar, they diverge. Culture is about relatively stable and deeply rooted norm-based behavior, while climate refers to more “superficial elements such as employees’ reactions, opinions and tendencies” ([37], p. 585). Therefore, the climate is a surface manifestation of culture [37]. Based on this, it is expected that, when employees perceive IMOC in a positive manner, this would have a positive impact on the organizational climate of CC. Moreover, when CC increases, because of IMOC, this should have a positive impact on employees’ WE. To the best of the authors’ knowledge, no previous research has examined this specific linkage. However, two arguments support this idea. First, IMOC as defined in this study focuses on managers’ norm-based behavior and their orientation toward satisfying the needs and wants of employees in their work roles. Consequently, the norm-based behavior of management, manifested in IMOC, models the “correct standard” of organizational climate for all employees such as the nature of CC in the organization. As such, and based on social learning theory [41], employees learn appropriate behavior from their managers as significant role models. When IMOC and CC are perceived as positive, this should significantly increase employees’ WE. The second argument supporting a linkage between the three variables can be found in the JD–R model. IMOC and CC are both based on the JD–R model, suggested as organizational-level resources in this study. However, as indicated in the aforementioned discussion, IMOC is critical for CC in the organization. Thus, the resources of IMOC serve as necessary inputs or “ingredients” to positively build and increase the CC resources. Consequently, when CC increases because of a positive impact of IMOC, this should lead to a positive increase in employees’ absorption, vigor and dedication, which are all embraced in the concept of WE. Thus, IMOC and CC are expected to work in tandem to strengthen nurses’ WE. The assumption about WE as a mediator between IMOC and CC can be formulated by this formal hypothesis:

- **Hypothesis 5b:** *The relationship between IMOC and WE is mediated by CC.*

##### WE as a mediator between IMOC, CC and SQC, TI and JS

As visualized in Fig. 1, WE is suggested to play a role as a mediator between IMOC and CC and SQC, TI and JS. The individual-level resource, WE, is expected to play a key role. Consequently, WE is assumed to function as the mediating mechanism that binds or ties the suggested variables together as a whole.

As noted in the previous discussion, IMOC and CC are closely related concepts. Because of their close relationship and association, it is reasonable to assume that the two concepts should work in the same direction depending on how employees appraise or perceive them. As Trus et al. noted, “culture and climate represent a social context … that constrains and promotes certain behaviors and interactions” ([42], p. 55). This study takes a positive perspective when studying the social context of IMOC and CC. Consequently, rooted in the assumption of the close relationship between IMOC and CC, it is expected that both are positive promoters of nurses’ WE. Moreover, when WE increases, because of nurses’ positive perception or appraisals of IMOC and CC, this should also lead to several positive job-related outcomes and thus an achievement of organizational goals such as increased SQC and JS and reduced TI of nurses in the organization.

The assumption of the mediating role of WE is summarized in these two final hypotheses:

- **Hypothesis 6:** *WE functions as a mediator between IMOC and a) SQC, b) TI and JS*
- **Hypothesis 7:** *WE functions as a mediator between CC and a) SQC, b) TI and JS*

Given the prevailing theoretical framework and its discussion of the conceptual model (Fig. 1), along with the hypotheses development in this study, a total of nine hypotheses have been proposed. These are summarized below in Table 1.

**Table 1:** Summary of the hypotheses in this study

Hypothesized relationships	
H1	*WE is significantly and negatively related to TI*
H2a	*WE is significantly and positively related to (JS)*
H2b	*The relationship between WE and TI is mediated by JS*
H3	*WE is positively related to SQC*
H4	*CC is positively related to WE*
H5a	*IMOC is positively related to WE*
H5b	*The relationship between IMOC and WE is mediated by CC*
H6	*WE functions as a mediator between IMOC and a) SQC, b) TI and JS*
H7	*WE functions as a mediator between CC and a) SQC, b) TI and JS*

Note: *WE* Work engagement, *IMOC* Internal market-oriented culture, *CC* Collaboration climate, *SQC* Service quality care, *TI* Turnover intention, *JS* Job satisfaction

## Methods

### Study design and settings

This study is part of a larger research project, and as such, the data for this study were collected from four public hospitals located in South East Norway. Initially, six public hospitals in the South East Norway region were invited to participate in the research project. Four of the six agreed to participate. Initial contact and all subsequent contact with the public hospitals was through hospital management. The management personnel of the public hospitals were presented a pitch on the research project, along with its aim, estimated time and required resources. With the help of hospital management, motivational letters that included information about the research project were sent to the employees of the four public hospitals. Information provided to the participants included knowledge about the aim of the research project, participants’ rights, length of the questionnaire, information on where to find the online questionnaire, and contact details of the research project leader(s). It is important to note that all information provided to the participants was approved by hospital management personnel, who then forwarded the information to their employees.

### Study participants

A total of 1104 nursing staff members received the questionnaire and 164 questionnaires were completed, giving a response rate of 14.8%. Previous research that focused on WE and used nursing staff as a sample encountered the challenge of increasing response rates when using convenience sampling and online surveys as data-gathering methods [43]. For example, Warshawsky [43] examined the influence of interpersonal relationships on nurse managers’ work engagement and proactive work behavior, and reported a response rate of 13%. Furthermore, the study of Slåtten and Svenkerud [2], which focused on nurses’ turnover intentions, reported a low response rate (15%) and argued that one reason pertains to the nature of work roles that fall under nursing staff, where most of their allotted time is assigned to patients, rather than at a computer desk. Consequently, because of the time limit of the project this study is affiliated with, combined with the nursing staff’s allotted time in voluntary participation in this study, a response rate of 14.8% can be considered adequate in this current cross-sectional study.

### Instruments

By combining capabilities and knowledge from academic experts and select hospital employees, several pretests were completed to ensure that the questionnaire was high quality. The reason for this is two-fold. First, the survey was completed in Norwegian, and as such, several workshops were required to ensure the quality of back-to-back translation. Second, to ensure the overall quality of the questionnaire, pretests were completed. The results of the various pretests helped us address the challenges pertaining to readability, redundancy or ambiguity. Consequently, the final questionnaire was modified and changed accordingly.

This study focuses on a total of six main instruments from the current literature that were used in this study: IMOC, CC, WE, SQC, TI and JS. All participants responded to the validated constructs using a seven-point Likert scale ranging from (1) strongly disagree to (7) strongly agree. In addition to the six main instruments, the study includes a section on demographic characteristics. It is important to note the all the survey instruments in this study are not under license. A supplementary file (see Additional file 1) details the questionnaire developed and used for this study.

In this study, JS refers to nursing professionals’ global assessment of their job. The items for JS are based on and adapted from Anaza and Rutherford [22]. The concept of IMOC refers to nursing professionals’ perception of managers’ norm-based behavior regarding three aspects of IMOC: (i) internal-market intelligence generation; (ii) internal-intelligence dissemination; and (iii) response to internal intelligence. The items for IMOC are based on and adapted from Gounaris [40]. The concept of WE in this study was defined as “a positive, fulfilling, work-related state of mind” ([14], p. 74) and represented three facets of state of mind: (i) absorption, (ii) vigor and (iii) dedication. The items for WE are based on Schaufeli et al. [14] and modified for this study. In this study, the concept of SQC relates to nursing professionals’ perceptions of their overall SQC. The items for SQC are based on Slåtten [44]. The concept of CC refers to nursing professionals’ perception of two aspects of interdepartmental collaboration: (i) level of conflict and (ii) level of connectedness. These two aspects are suggested in previous research. The items for CC are based on the work of Kohli and Jaworski [31, 32] and modified for this study. The concept of TI reflects frontline employees’ cognitive reflection about whether they should stay with the organization that employs them. The items for TI are based on Boshoff and Allen [45].

### Data collection procedure

After the pretests were completed, the final questionnaire was distributed by the management of the four hospitals to their nursing staff by e-mail. Data were collected through the online software program Checkbox and were then analyzed using the Stata software. In the process of data collection, several reminders to participate in the study were sent out by the hospital management.

### Data analysis

Structural equation modeling was applied to explore the relationships between the constructs. The first step was to assess the measurement model (consisting of reflective latent constructs); step two tested the structural model. The estimation used the “sem” package in Stata [46]. Testing of the mediation hypotheses was conducted using the “medsem” package in Stata [47], following the approach proposed by Zhao et al. [48].

The measurement model was assessed by examining several criteria: goodness-of-fit indices (SRMR, RMSEA, CFI, TFI); indicator reliability (item loading); latent construct reliability (Raykov’s reliability coefficient); convergence validity (average variance extracted (AVE; all AVE values should be larger than the squared correlations among the latent constructs)); and discriminant validity. Convergent and discriminant validity make up the construct validity. The “rules of thumb” used in Tables 2 and 3 are based on Mehmetoglu and Jacobsen [46].

**Table 2:** Personal characteristics of the study sample (*N* = 164)

		%
Sex:	Female	93.3
	Male	6.7
Work as:	Nurse	43.9
	Specialist nurse	49.4
	Midwife	6.7
Employed:	fewer than 5 years	20.7
	6–10 years	15.3
	more than 10 years	64.0
Part-time or full-time:	part-time job	50.6
	full-time job	49.4
Age:	younger than 40 years	34.8
	41–50 years	29.9
	older than 50 years	35.3

**Table 3:** Results of the measurement model for the constructs internal market-oriented culture, collaboration climate, work engagement, service quality of care, turnover intention and job satisfaction

Construct Question items	Loading	RRC	AVE
“Rule of thumb”	> 0.4	> 0.7	> 0.5
***Internal market-oriented culture (IMOC)***		0.962	0.679
Employees have the opportunity to discuss their needs with management.	0.831*		
Training is seen in the context of individual needs.	0.738*		
Management is being encouraged to meet to discuss issues concerning their employees.	0.821*		
I believe management will spend time talking to me when I need it.	0.765*		
Management understands the needs of employees.	0.898*		
Management wants employees to enjoy their work.	0.861*		
I believe that management shows a sincere interest in any problems I have doing my job.	0.896*		
I believe that management understands that personal problems may affect my performance.	0.776*		
The division’s policies help meet employees’ individual needs.	0.856*		
Management meets regularly to discuss issues related to employees’ challenges.	0.823*		
If an employee from my department is faced with a serious problem, the managers in my division are notified immediately.	0.681*		
Management works hard to accommodate employees’ needs.	0.907*		
***Collaboration climate (CC)***		0.892	0.594
It is easy to talk with everyone in my division, regardless of rank or position.	0.716*		
Employees like interacting with those from other departments.	0.706*		
There is little conflict between departments in the divisions.	0.743*		
Employees from different departments are available to help each other when needed.	0.704*		
There is open communication between the departments.	0.897*		
The departments in our division cooperate well with each other.	0.836*		
***Work engagement (WE)***		0.851	0.671
I am so into my job that I lose track of time.	0.720*		
This job is all-consuming; I am totally into it.	0.936*		
I put my soul into my job.	0.770*		
***Service quality of care (SQC)***		0.928	0.810
In my view, I offer good patient service.	0.933*		
In my view, I offer patient services of very high quality.	0.879*		
In my view, I offer the patients a high degree of service.	0.886*		
***Turnover intention (TI)***		0.874	0.697
I often think about resigning from my job.	0.850*		
It would not take much to make me resign from my job.	0.817*		
I will probably be looking for another job soon.	0.837*		
***Job satisfaction (JS)***		0.933	0.782
If a good friend of mine was interested in a job like mine in this organization, I would strongly recommend it.	0.865*		
My job is the sort of job I wanted when I took it.	0.896*		
If I had to decide all over again whether to take a job in this organization, I would.	0.868*		
Overall, I am satisfied with my current job.	0.908*		

* *p* < 0.05. *RRC* Raykov’s reliability coefficient. *AVE* Average variance extracted

The structural model was assessed with the same goodness-of-fit measures as the measurement model. The structural model’s reported path coefficients are standardized values, which range between − 1 and 1. The closer a path coefficient is to ±1, the stronger is the relationship.

### Ethical considerations

The final questionnaire was submitted for approval to Norwegian Social Science Data Services (NSD), project number 42091, to ensure compliance with the research ethics set by NSD. In addition, participants had to consent to the voluntary participation prior to the commencement of the survey. As already mentioned, all contact with the participants occurred through the hospital management. To ensure participants’ anonymity, information that could identify the participants, such as IP addresses, was deleted upon the completion of the survey.

## Results

### Characteristics of the study sample

Table 2 shows the personal characteristics of the study sample. Most responses were received from employees who worked as nurses or specialist nurses. A large portion of nursing professionals had significant experience. Of the total sample, 64% had worked in the investigated hospitals for more than 10 years. About 50% worked full-time. About 35% were under 40 years of age, about 30% were 41–50 years of age, and about 35% were older than 50 years of age.

### Measurement model

Table 3 shows that the reliability and validity measures were all within the rules of thumb, and Table 4 shows that the goodness-of-fit indices were also within the commonly accepted thresholds, indicating that a sound measurement model was established.

**Table 4:** Summary of the fit statistics of the measurement and structural models

Model	*χ*^2^	d.f.	RMSEA	CFI	TLI	SRMR
Fit criteria (“Rule of thumb”)			< 0.1	> 0.9	> 0.9	< 0.1
Measurement model	683.85	419	0.062	0.941	0.934	0.055
Structural model	719.88	427	0.065	0.934	0.928	0.085

*d.f.* degrees of freedom, *RMSEA* root mean square error of approximation, *CFI* comparative fit index, *TLI* Tucker–Lewis index, *SRMR* standardized root mean square residual

### Structural model

**Fig. 2:**
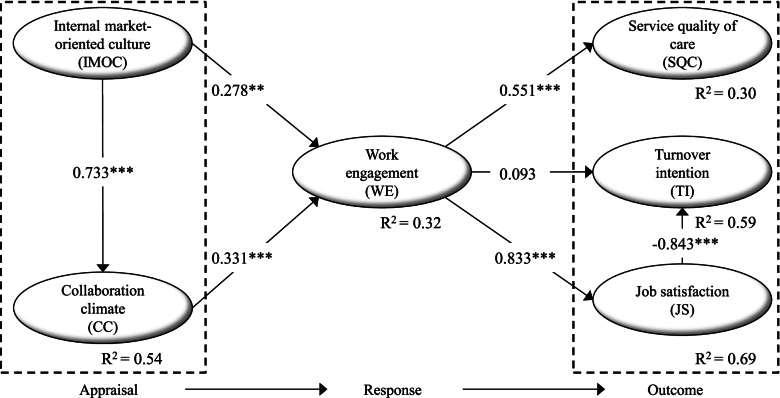
Results of the structural model to analyze the precursors and outcomes of work engagement. Standardized coefficients (*** *p* < 0.01, ** *p* < 0.05)

Table 4 indicates that the goodness-of-fit indices for the structural model are also within the commonly accepted thresholds. Figure 2 presents the standardized path coefficients and their significance level. We found that IMOC and CC had a positive and statistically significant and about equal effect on WE, and thus support hypotheses H_5a_ and H_4_. WE had a large and statistically significant effect on both SQC and JS, and thus supports H_3_ and H_2a_. The relationship between WE and TI was not significant and H_1_ was not supported. The model explains 32% of the variance in WE, 30% of the variance in SOC, 59% of the variance in TI and 69% of the variance in JS.

Using mediation analysis, Table 5 shows that the indirect effect of job satisfaction on the relationship between WE and TI is significant (β = − 0.501), implying a full mediator effect and support for H_2b_. CC has a significant indirect effect (β = 0.207) and a partial mediation effect between IMOC and WE, and thus H_5b_ is supported.

**Table 5:** Mediator analysis. Standardized direct, indirect and total effects

Hypothesis	Effect^a^	Mediator	Direct effect^a^	Indirect effect	Total effect	Mediator effect^b^
H_2b_	WE → TI	JS	0.085	−0.501***	0.416	Full
H_5b_	IMOC → WE	CC	0.275**	0.207**	0.482	Partial
H_6a_	IMOC → SQC	WE	−0.155	0.141**	0.014	Full
H_6b_	IMOC → TI	WE	−0.179	0.023	−0.156	No
H_6c_	IMOC → JS	WE	0.111	0.178**	0.289	Full
H_7a_	CC → SQC	WE	0.211	0.145**	0.356	Full
H_7b_	CC → TI	WE	0.069	0.024	0.093	No
H_7c_	CC → JS	WE	0.223***	0.183**	0.406	Partial

Notes: *IMOC* Internal market-oriented culture, *CC* Collaboration climate, *WE* Work engagement, *SQC* Service quality of care, *TI* Turnover intention, *JS* Job satisfaction

** *p* < 0.05, *** *p* < 0.01 are significance levels

^a^ The direct effects (the links *X* → *Y*) in our basic structural model (Fig. 2) are almost identical in our model for mediation analysis

^b^ We used the bootstrapping test of Zhao et al. [48] to test mediation. Briefly, this approach tests, through bootstrapping, whether the direct and indirect effects are statistically significant, and the combination of these two tests decides if there exists no, partial or full mediation

We tested a total of six mediator effects of WE, and our findings (Table 4) show in general that WE is a strong mediator. WE has a significant indirect (β = 0.207) and a full mediation effect on the relationship between IMOC and SQC, supporting H_6a_. WE does not mediate the relationship between IMOC and TI, and thus does not support H_6b_. However, WE intervenes between IMOC and JS (with an indirect effect of β = 0.178), supporting H_6c_. WE fully mediates between CC and SQC (with an indirect effect of β = 0.145), supporting H_7a_. The relationship between CC and TI has no significant indirect effect and thus is not mediated by WE, indicating no support for H_7b_. Lastly, WE partially mediates the relationship between CC and JS, with an indirect effect of β = 0.183, supporting H_7b_.

## Discussion

To the best of our knowledge, this is the first study to undertake a comprehensive examination of nursing professionals’ WE using several direct and indirect effects associated with WE, in the same study. By doing so, it contributes to both extending and deepening research on this topic in healthcare services research. Specifically, it responds directly to Keyko et al.’s call for further research focused on “the importance … of … work engagement” ([9], p. 161).

The contributions in this study can be divided into three sub-contributions. First, this study embraces several different types of organizational outcomes associated with nursing professionals’ WE. Specifically, it includes both patient-related outcomes (represented by SQC), job-related outcomes (represented by JS) and employer-related outcomes (represented by TI). In doing so, this study provides new insight into how nursing professionals’ WE is associated with a variety of desired goals in healthcare organizations. Second, it contributes to revealing how nursing professionals’ perceptions of organizational culture (represented by IMOC) and organizational climate (represented by CC) act as a precursor to their WE. Thus, it enhances our understanding of how potential strategies related to the individual-level resource of WE can be cultivated and managed by the two organizational-level resources (referring to IMOC and CC). Third, consistent with our overall aim and focus, the study has undertaken an extensive test of the centrality or role of healthcare professionals’ WE in healthcare organizations. Specifically, using complex statistical tests and analysis, it examines whether WE operates as a full or partial mediator between organizational-level resources (IMOC and CC) and the three organizational goals (SQC, TI and JS) included in the study. As such, the study provides a comprehensive contribution regarding the true “value of … work engagement” ([9], p. 161) for healthcare organizations.

In line with much of the previous research, the individual-level resource of nursing professionals’ WE is defined as “a positive, fulfilling, work-related state of mind” ([14], p. 74). Previous research has suggested that this positive state of mind embraced in WE is generally able to “improve … organizational outcomes” ([16], p. 2). However, when considering the domain of healthcare service research, there has been a specific lack of studies on the outcomes of WE of nursing professionals and “customer” or patient-related outcomes. Regarding this, in their review of WE, Keyko et al. [9] commented that “one surprising finding of this review was the lack of research on patient-related outcomes.” In contrast to the lack of this focus in previous research, this study contributes by including SQC as a patient-related outcome. The findings identify a linkage between WE and SQC (β = 0.551). WE explains 30% of the variance in SQC. As such, it provides empirical support for the positive impact of WE. Specifically, it contributes to revealing the value of having an engaged workforce and how healthcare organizations can capitalize on having an engaged workforce because it is capable of substantially increasing patients’ experience and perception of service quality.

This study also reveals that the value of the WE of nursing professionals is not restricted only to having an impact on the patients’ perception of SQC of the healthcare organizations. The results show that WE among nursing professionals also has a positive impact on their internal values, referring to their JS, which in this study is defined as to what “extent employees like their jobs” ([21], p. 13). WE is strongly linked to the JS of healthcare professionals (β = 0.833). WE explains almost 70% (*R*^2^ = 0.69) of the variance in nursing professionals’ JS, which is substantial. Consequently, this implies that when leaders in health organizations know the levels of WE of their nursing professionals, it indicates whether they are satisfied with their job. Thus, WE can function as an organizational “thermometer” that leaders of healthcare organizations can actively use to identify (negative or positive) trends in healthcare professionals’ experience with their work-related conditions.

The results from this study show that JS is also linked to the TI of nursing professionals (β = − 0.843) explaining almost 60% (*R*^2^ = 0.59) of the variance in TI. Specifically, JS was found to act as a mediator between WE and TI. No significant direct effect of WE on TI was identified. Most often, WE is linked individually to both TI [6, 19, 20] and JS [9, 23]. Consequently, this lack of significant findings differs from previous research. However, in contrast to most previous research, this study undertakes a considerably more complex and comprehensive test of how WE is potentially associated with TI, suggesting JS as a mediator. Previous research on this is limited because it has only theoretically conceptualized JS as a mediator between WE and TI (e.g., [16]). To the authors’ knowledge, no study in health services research has empirically examined whether the JS of nursing professionals mediates the relationship between WE and TI. Consequently, this study contributes to an alternative and more nuanced understanding of how WE is linked to TI. Although WE and JS are conceptually different, they share a commonality because both focus on nursing professionals’ perception of aspects of their work role. By contrast, TI is about the nursing professionals’ perception of their employer and organization and whether they evaluate this as a great place to work. Consequently, TI is related to a “decision … process” ([18], p. 23). Based on these distinctions, it is reasonable to assume that JS, more so than WE, is an integral part of nursing professionals’ decision process related to TI. Therefore, it is both natural and more logical, as this study also has revealed, that JS functions as a mediator between WE and TI. Accordingly, JS of nursing professionals, stemming from their level of WE, is linked to individuals’ decision processes about whether they should continue or stop working for an organization that they are employed in at present.

As noted previously in this paper, WE as an individual-level resource is considered and described as something that is not static but dynamic. This dynamic aspect implies that the WE of nursing professionals is potentially changeable or manageable. This study contributes to empirically demonstrating that this assumption seems to be correct. Both IMOC and CC, representing two types of organizational-level resources, can positively change or “manage” the WE of nursing professionals. The findings also contribute to revealing a close association between organizational culture (refer to IMOC) and organizational climate (refer to CC). Simultaneously, it also reveals how IMOC has an impact on WE through CC. IMOC and CC explain more than 30% (*R*^2^ = 0.32) of the variance in WE. Although the concepts of organizational culture and organizational climate, in general, have been linked to WE in previous research [6, 12, 34], very little research has been undertaken within the domain of healthcare. To the authors’ knowledge, this is the first study in healthcare services research that includes and examines both IMOC and CC in the same study. By doing this, it reveals a multifaceted pattern of how IMOC and CC are linked to the WE of nursing professionals. As such, the study directly responds to Slåtten et al.’s ([12], p. 177) call for more research on IMOC and WE. Furthermore, it also contributes to increasing the amount of research on organizational culture studies in healthcare settings. Mesfin et al. recently noted that “there are only a few studies [on organizational culture] in a healthcare setting” ([49], p. 3). In addition to these calls for more research, this paper also extends previous research on WE in another manner. Specifically, it examines the extent of centrality or strength of WE when considering two organizational-level resources, IMOC and CC, and the actual achievement of organizational goals SQC, TI and JS. The findings reveal that the WE of nursing professionals mediates all the suggested relationships in the proposed model, with the exception of TI. This finding is a significant contribution to both research on WE in health services and specifically to identifying the role that the WE of nursing professionals seems to play for the achievement of desirable organizational goals in health organizations. Consequently, leaders and managers of healthcare services organizations should continuously have the WE of their nursing professionals on their meeting agendas because it seems to be a core variable and strategic key tool to enable multiple desirable organizational outcomes. In summary, this study has important implications for how to handle one of the biggest challenges facing organizations today, as described in Kaye and Jordan-Evans: “The challenge today is not just retaining talented people, but fully engaging them, capturing their minds and hearts at each stage of their work lives” [8].

## Limitations and future research

Although this study contributes to health services research by examining several interesting and important precursors and outcomes associated with WE, more research on WE is required in the future.

First, this study employed cross-sectional survey data with convenience sampling from 164 nursing staff in four public hospitals in South East Norway. Although this study followed the recommendations of Levine [50] in conducting a cross-sectional analysis, there are various methodological limitations that might offer opportunities for future research in examining nursing professionals’ WE. For example, the data used in this study were collected at one point in time, in a specific region in Norway. Furthermore, this study collected data using an online survey. In addition, although this study ensured participant anonymity, participants in this study reported on their perceived WE through self-reported measures. These methodological limitations limit the generalizability of the study’s result to other public hospitals. Specifically, the relatively small sample size means that the results of this study should be interpreted with caution. In addition, the collected data may suffer from self-selection bias [51], self-report issues [52], share response bias [53] and issues pertaining to reversed causality [45]. Future research should test for causality of the studied relationships, collect data at various points in time, or adopt a longitudinal data collection method, broaden the sample and collect data from various regions and countries.

Second, this study limited its focus on the WE of health professionals from the perspective of individuals. While this has been the most conventional way to analyze WE, other levels could be included. One such approach would be to focus on WE from an organizational perspective and what the literature refers to as collective work engagement. This implies a shift in focus from “I” or individual engagement to “We” or organizational climate engagement. In general, limited research has been undertaken on collective engagement [54]. To the authors’ knowledge, no study has been undertaken that has focused specifically on aspects associated with health professionals’ collective engagement. Research outside health services research, but within knowledge-based firms, has revealed that collective engagement is positively associated with areas such as relationship learning, firms’ innovative capability and employee commitment [55]. Given these positive outcomes, it is strongly recommended that future research in health services also includes a focus on collective engagement. One concrete suggestion of this study is, and as also suggested in Slåtten et al. [12], to “examine whether and how IMOC is connected to … collective engagement” ([12], p. 177). Such a focus, among several other potential factors, would contribute clearly to broadening our understanding of the concept of WE.

Third, according to Mesfin et al., “a good hospital culture leads to better individual performance” ([49], p. 3). This study limits its focus to study organizational culture represented by the concept of IMOC. Future research could include other potential types of organizational culture. One concrete suggestion would be to include one or more of the four organizational culture typologies, that is, 1) clan, 2) adhocracy, 3) market and 4) hierarchy, as mentioned in Cameron and Quinn, which is known as “the competing value framework” [56]. Studying these organizational culture typologies and their association with potential antecedents or outcome variables extends our understanding of what type of culture can develop and manage employees’ WE for the achievement of organizational goals in healthcare organizations.

Fourth, this study only includes the concepts of culture (referring to IMOC) and climate (referring to CC) as drivers of WE. Consequently, we do not examine the impact of leadership, which is a limitation of the study. Although one could argue that both IMOC and CC are affected by leadership, future research should clearly include leadership as a precursor to WE. The literature provides a range of potential leadership perspectives and styles that could be included, such as transformational leadership, ambidextrous leadership, transactional leadership, empowering leadership and leadership autonomy support. However, one concrete suggestion would be to include the four leadership styles mentioned in the Path–Goal Theory Conceptual Framework, that is, 1) directive, 2) achievement-oriented, 3) participative and 4) supportive [57]. Similar to the suggestion to include other types of organizational culture, examining a range of leadership styles would extend our insight into “what to do” in the best possible way to manage health professionals’ WE for the achievement of organizational goals.

## Conclusion

In conclusion, this study examines whether the WE of nursing professionals is a core factor in the achievement of the preferred organizational goals of healthcare organizations. WE was found to be correlated both to nursing professionals’ level of SQC and their JS. WE and TI are mediated by JS. Based on these findings, leaders and managers of hospital organizations should manage nursing professionals’ WE, focusing on organizational culture and climate. In conclusion, the WE of nursing professionals is important because it enables hospitals to achieve multiple desirable outcomes.

## Supplementary Information

**Additional file 1.**

## Data Availability

The datasets used and/or analyzed in this study are available from the corresponding author on reasonable request.

## Abbreviations

IMOC: Internal market-oriented culture
CC: Collaboration climate
WE: Work engagement
SQC: Service quality of care
TI: Turnover intention
JS: Job satisfaction
SEM: Structural equation modeling
SRMR: Standardized root mean square residual
RMSEA: Root mean square error of approximation
CFI: Comparative fit index
TLI: Tucker–Lewis index
RCC: Raykov’s reliability coefficient
AVE: Average variance extracted
SQ: Service quality
NSD: Norwegian Social Science Data Services
