# Supplementary material for: Precursors and outcomes of work engagement among nursing professionals—a cross-sectional study
Source: BMC Health Serv Res. 2022 Jan 4;22:21. doi: 10.1186/s12913-021-07405-0 (PMC8725263; doi:10.1186/s12913-021-07405-0)
Supplement: Supplementary file 1 — Additional file 1. [file 12913_2021_7405_MOESM1_ESM.docx]

**ADDITIONAL FILE 1: ELECTRONIC QUESTIONNAIRE DEVELOPED FOR THIS STUDY**

Sex:

Male

Female

Age:

younger than 40 years

41 and 50 years

older than 50 years

Part-time or full time:

Part-time job

Full-time job

Employed:

Fewer than 5 years

6-10 years

More than 10 years

I work as:

Nurse

Specialist nurse

Midwife

On a 7-point scale from 1 (Strongly disagree) to 7 (Strongly agree), please rate how you agree or disagree with the following statements:

Employees have the opportunity to discuss their needs with management.

Training is seen in the context of individual needs

Management is being encouraged to meet to discuss issues concerning their employees

I believe management will spend time talking to me when I need it

Management understands the needs of employees

Management wants employees to enjoy their work

I believe that management shows a sincere interest in any problems I have doing my job

I believe that management understands that personal problems may affect my performance

The division’s policies help meet employees’ individual needs

Management meets regularly to discuss issues related to employees’ challenges

If an employee from my department is faced with a serious problem, the managers in my division are notified immediately

Management works hard to accommodate employees’ needs

It is easy to talk with everyone in my division, regardless of rank or position

Employees like interacting with those from other departments

There is little conflict between departments in the divisions

Employees from different departments are available to help each other when needed

There is open communication between the departments

The departments in our division cooperate well with each other

I am so into my job that I lose track of time

This job is all-consuming; I am totally into it

I put my soul into my job

In my view, I offer good patient service

In my view, I offer patient services of very high quality

In my view, I offer the patients a high degree of service

My job is the sort of job I wanted when I took it

Overall, I am satisfied with my current job

I often think about resigning from my job

It would not take much to make me resign from my job

I will probably be looking for another job soon
